# The effect of bright light therapy in migraine patients with sleep disturbance: A prospective, observational cohort study protocol

**DOI:** 10.3389/fnagi.2022.1041076

**Published:** 2023-01-19

**Authors:** Tsung-Hsing Lin, Cheng-Chia Yang, Shih-Yu Lee, Ching-Mao Chang, I-Ju Tsai, Cheng-Yu Wei, Chun-Pai Yang

**Affiliations:** ^1^Department of Emergency Medicine, Kuang Tien General Hospital, Taichung, Taiwan; ^2^Department of Healthcare Administration, Asia University, Taichung, Taiwan; ^3^Biotechnology Health and Innovation Research Center, Hungkuang University, Taichung, Taiwan; ^4^College of Nursing, Hungkuang University, Taichung, Taiwan; ^5^Center for Traditional Medicine, Taipei Veterans General Hospital, Taipei, Taiwan; ^6^Faculty of Medicine, National Yang Ming Chiao Tung University, Taipei, Taiwan; ^7^Institute of Traditional Medicine, National Yang Ming Chiao Tung University, Taipei, Taiwan; ^8^Department of Neurology, Kuang Tien General Hospital, Taichung, Taiwan; ^9^Department of Exercise and Health Promotion, College of Kinesiology and Health, Chinese Culture University, Taipei, Taiwan; ^10^Department of Neurology, Chang Bing Show Chwan Memorial Hospital, Changhua, Taiwan; ^11^Ph.D. Program in Translational Medicine, National Chung Hsing University, Taichung, Taiwan

**Keywords:** migraine, light therapy, circadian rhythms, PSG, neuropeptides, wrist actigraph

## Abstract

**Background:**

Migraine is a common disabling disorder, and its substantial burden is associated with a considerable negative impact on the patients’ quality of life. Moreover, aging patients with migraine have more cognitive complaints. Additionally, the elderly are more likely to have sleep disturbances, which may also predict the risk of incident dementia. Migraines are reported to be closely associated with sleep and circadian rhythms. Sleep disturbance is a well-known trigger for migraine episodes; moreover, shift work or jet lag reportedly triggers some migraines. The hypothalamus is thought to be the migraine generator; sleep and circadian activity rhythm are also controlled by the hypothalamus. Evidence suggests an influence of both sleep and circadian system on migraine. Previously, light therapy has been show to stabilize sleep architecture and further improve insomnia related to circadian rhythm disorders. However, the beneficial effect of light therapy on migraine with sleep disturbance has not yet been determined. We aim to explore the effects of light therapy for migraine combined with sleep disturbance.

**Methods and analysis:**

This project is a 2-year, randomized, double-blind, placebo-controlled clinical trial. The study design includes a 4-week monitoring period (baseline and pretest), a 4-week treatment period, and a posttest. The study participants will undergo assessments on headache frequency and severity and subjective and objective (wrist actigraphy and polysomnography) sleep disturbances, and quality of life and a series of blood tests for serum biomarkers.

**Discussion:**

This study will establish evidence-based alternative medicine for the preventive effect of bright light therapy in migraine patients with sleep disturbances. Moreover, our data will be useful to comprehend the biochemical mechanism of light therapy in migraine prevention.

**Clinical Trial Registration**: ClinicalTrials.gov, identifier NCT04890691.

## Introduction

Over 1 billion people experience migraine, with approximately three times as many women as men suffering (Ashina et al., 2021a,b). The incidence of migraine in Taiwan is 9.3%, with the most common age group being young adults aged 35–55 years, and the ratio of women to men being approximately 3:1 (Wang et al., 2000). Migraine is one of the five most disabling and most burdensome conditions in women (Stovner et al., 2007; Steiner et al., 2018). Generally, if migraine is undertreated, it not only causes extreme discomfort to patients, but also reduces their quality of life (QOL) and work productivity, increases absenteeism, and places a significant burden on the healthcare system (Burstein et al., 2015). Additionally, migraine patients also have many comorbidities, including mental illnesses (e.g., depression, anxiety), sleep disorders, and pain in other body parts. Migraine patients often complain of lack of sleep before and during a migraine attack, whereas good sleep is helpful for migraines. Lack of sleep is often cited as one of the causes of migraine. Apart from the lack of sleep triggering migraines, too much sleep or circadian rhythm disturbances, such as shift interference, can also trigger migraine attacks (Vgontzas and Pavlovic, 2018). These phenomena suggest that migraines are not only related to sleep but also sleep rhythm disorders. The hypothalamus in the brain is responsible for circadian rhythm and sleep, and it is confirmed as the migraine generator (Burstein et al., 2015); thus, we hypothesized that day and night rhythm regulation or stabilization can reduce migraine attacks.

Although the link between migraine and sleep has been documented for decades, its physiological mechanism is still unclear. Migraine exhibits menstrual and circadian rhythms, and individuals with migraine may have a defect in chronobiologic synchronizing systems (Silberstein, 2008). Women seem more vulnerable to the effects of sleep disturbances than men because of both physiological and psychosocial factors. For example, shifting of hormone levels, including those occurring with puberty, menstruation, and perimenopause, may modulate circadian rhythms among women (Brandes, 2006), and may be responsible for substantive changes in the nature of women’s sleep. Circadian rhythms affect sleep and wakefulness in humans, and exposure to sunlight helps stabilize and regularize the circadian rhythms (Lindskov et al., 2022).

Migraine is caused by the activation of the trigeminovascular system, resulting in the release of pain-producing nerve-inflaming substances around the nerves and blood vessels in the head and neck (Burstein et al., 2015). Migraine is reportedly initiated at the same location as the center that controls the circadian rhythm, the hypothalamus, which may explain why sleep disturbances are both a precipitating factor and common comorbidity in migraine (Baksa et al., 2019; Tiseo et al., 2020). The pathogenesis of migraine is extremely complex, and the “trigeminovascular theory” is the main theory currently recognized (Noseda and Burstein, 2013). This theory holds that activation of trigeminal nerve endings from around the meningeal blood vessels causes localized nerve inflammation, which mainly includes the following two neuropeptides: calcitonin gene-related peptide (CGRP) and pituitary adenylate-cyclase-activating polypeptide (PACAP). These two peptides will further cause the activation of the trigeminal nucleus and stimulate the cerebral cortex to produce headaches (Amin et al., 2016; American Headache Society, 2019).

One of the main treatment methods of migraine is to reduce the triggering factors, and sleep disturbance is among the common triggering factors of migraine. However, few sleep interventions have been used to reduce the frequency and severity of migraine attacks. Bright light therapy (BLT) can reportedly improve nighttime sleepiness due to circadian rhythm or neuro-psychiatric disorders and sleep duration and reduce daytime sleepiness (Burkhalter et al., 2015; Faulkner et al., 2019); however, the effect of BLT in migraine patients with sleep disturbance is uncertain. Particularly, light has been viewed as a trigger for migraine, but a recent review reported that migraine patients are not photophobic to all wavebands of the light spectrum (Artemenko et al., 2022). Thus, light therapy should be an innovative approach. Additionally, the mechanism of the link between sleep disturbance and migraine is still not clearly understood, and understanding the pathological mechanism is helpful for future clinical care and further research. Therefore, we aim to examine a BLT intervention designed to reduce migraine in women by improving the sleep and circadian activity rhythms. Subsequently, we will test a BLT intervention as part of the sleep hygiene bundle designed to reduce migraine attacks in women by improving sleep and circadian activity rhythms.

### Objectives

The specific goals of this prospective study are to (1) compare the effects of BLT (green light and sleep hygiene) with an attention control treatment (placebo light and sleep hygiene) on selected sleep variables [total nocturnal sleep time, wake after sleep onset (WASO), sleep efficiency, daytime sleepiness], migraine conditions (frequency and severity), and QOL (mental health) from baseline to 1 month after beginning the intervention; and (2) explore the light therapy’s molecular and biochemical effects on migraine prevention.

## Materials and methods

### Study design

**Figure 1:**
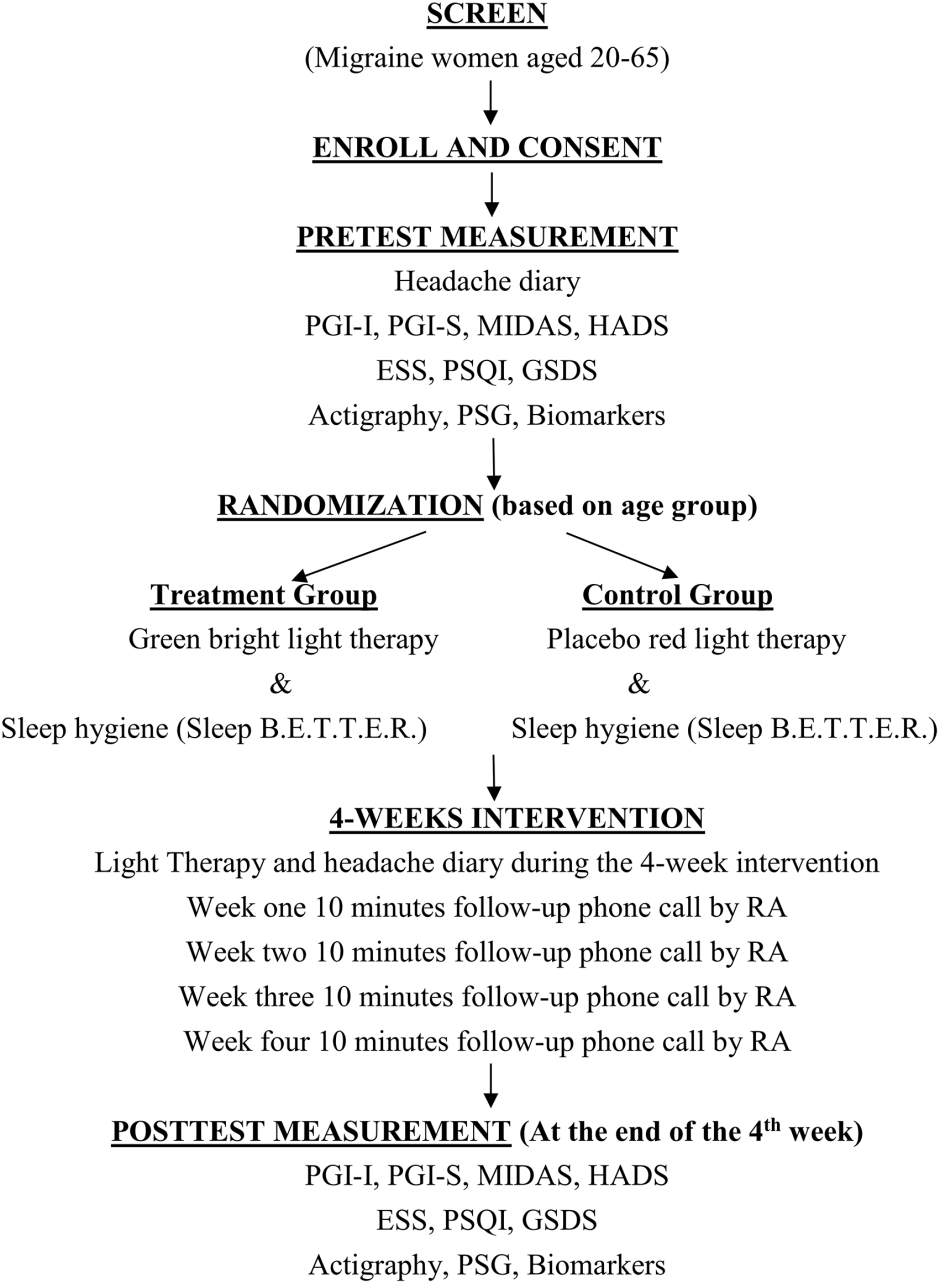
Randomized pretest–posttest design.

This parallel, randomized, double-blind, placebo-controlled clinical trial will be conducted at a teaching hospital in the middle part of Taiwan. The sample will consist of 60 women diagnosed with migraine and sleep disorders. After a 1:1 random assignment, 30 women will be assigned to light therapy and sleep hygiene education, and the other 30 women will be assigned to placebo light and sleep hygiene education for a 4-week clinical trial (Figure 1).

### Inclusion and exclusion criteria

The inclusion criteria are as follows: diagnosed with migraine according to the International Classification of Headache Disorder 3rd edition (ICHD-3); aged 20–65 years; with sleep disorders (PSQI >5 points); willing to participate in activity monitoring (wrist recorder) during the day and night for 9 consecutive weeks, and one night in the sleep room for polysomnography, totaling to two time periods (before and after treatment); willingness to have blood drawn twice; not allergic to metals (a contraindication to wearing wrist actigraph); and not currently participating in other interventional trials.

The exclusion criteria are as follows: non-migraine headaches (e.g., tension-type or secondary headaches, among others); major head trauma in the past; alcoholism within 1 year; (4) pregnant or breastfeeding women; (5) cannot cooperate with the test progress; (6) allergic to light; and (7) taking beta-blockers, antiepileptic drugs, calcium ion blockers, antidepressants, onabotulinumtoxinA, CGRP monoclonal antibody, and hormone preparations in the past month.

### Interventions

Our study has a two-component intervention package, including a 30-min daily BLT (green and red lights for the treatment and control groups, respectively) and a sleep hygiene educational package for 4 weeks. The bright light visor (Feel Bright Light Model 100, Physician Engineered Products, Fryeburg, ME) will be used for the treatment group; however, the visor delivers blue–green light, thus we blocked the blue light and remind the participants to use the green light to prevent potential damage to eyes, especially the elderly. The unit weighs approximately 1.7 ounces, is comfortable to wear, and is powered by a rechargeable battery, allowing participants to move freely for their daily work. Participants will undergo 30 min of BLT within an hour of waking up for 4 weeks. A red-light visor (Physician Engineered Products, Fryeburg, ME), an attention control strategy, that does not have any therapeutic effect will be used for the control group. Both groups will receive a sleep hygiene educational booklet.

Sleep hygiene is fundamental to improving sleep and is considered an important intervention to promote sleep (McCurry et al., 2004). Sleep B.E.T.T.E.R. will be included in this educational package. This booklet includes information on normal sleep and promoting the homeostatic drive for sleep using the following six basic guidelines: bedroom environment, regular daily exercise, reducing tension and stress, time trying to sleep, eating and drinking, and maintaining regular day–night rhythms.

### Participant recruitment

A research assistant (RA) under a neurologist’s supervision will screen the visiting log at the research site’s migraine clinic to identify potential participants. Then, the RA will approach the women and describe the study. The opportunity will be given to the women to privately consider the project, and a later contact will be made to answer questions and enroll the women interested in participating.

### Data collection

Apart from the demographic form, psychometric sound instruments will be used in the study, which are described below (Table 1).

**Table 1:** Measurement schedule.

	Baseline	Treatment and data collection timepoints			
Timepoint	Pretest	1st week	2nd week	3rd week	4th week
Enrolment					
Eligibility screening	X				
Informed consent	X				
Demographics	X				
Medical records	X				
MEASUREMENTS					
4-week headache diary	X X X X	X	X	X	X
PGI-I	X				X
PGI-S	X				X
MIDAS	X				X
HADS	X				X
ESS	X				X
PSQI	X				X
GSDS	X				X
7-day wrist actigraphy	X				X
7-day sleep diary	X				X
Overnight PSG	X				X
Blood sample	X				X
Weekly phone call		X	X	X	X

PGI-I, Patient Global Impression of Improvement; PGI-S, Patient Global Impression of Severity; MIDAS, Migraine Disability Assessment; HADS, Hospital Anxiety and Depression Scale; ESS, Epworth Sleepiness Scale; PSQI, Pittsburgh Sleep Quality Questionnaire; GSDS, General Sleep Disturbance Scale; PSG, Polysomnography.

#### Headache diaries

This diary includes headache days, duration, intensity, frequency, location, characteristics, use of pain medication, accompanying symptoms and triggers, among others. Each diary covers 28 days.

#### Detection of headache-related scales

Patient Global Impression of Improvement (PGI-I) is an assessment to measure disease improvement (Guy, 1993). PGI of Severity (PGI-S) will be used to measure the severity of migraine (Guy, 1993). The Migraine Disability Assessment (MIDAS) (Stewart et al., 1999) will be used to assess migraine-related disabilities in different areas of life in the past 3 months.

#### The hospital anxiety and depression scale

This will be used to evaluate the depression and anxiety of chronic migraine patients (Zigmond and Snaith, 1983). Cut-off points are ≥ 10 and ≥ 13 for depression and anxiety, respectively.

#### Epworth sleepiness scale

The study participant’s daytime sleepiness will be measured with ESS (Johns, 1991).

#### Pittsburgh sleep quality questionnaire

This records the sleep status of the past month, including the following six sub-items: sleep quality, sleep onset time, sleep hours, sleep efficiency, sleep disturbance, use of sleep medication, and daytime activity. It will be filled in by self-assessment, with a total score of > 5 indicating poor sleep quality (Buysse et al., 1989).

#### General sleep disturbance scale

This questionnaire utilizes a weekly sleep rating scale of 0–7, which includes 21 items related to frequency, in the past week, quality of sleep, quantity of sleep, fatigue and wakefulness at work, and use of sleep-promoting substances (Lee, 1992; Lee and DeJoseph, 1992; Lee, 2007).

#### Wrist actigraphy (Mini Motionlogger Actigraphy, AMI, Ardsley, NY)

A lightweight watch-lime motion sensor monitor will be used to collect objective sleep data, including total nocturnal and day sleep time (TST), WASO, and circadian activity rhythms for 7 consecutive days. Various investigators in the past have demonstrated a very high correlation (*r* = 0.93–0.99) between the two measures of total sleep time during the night and measures of wakefulness using the sleep polysomnography (PSG) (Jean-Louis et al., 1996; Ancoli-Israel et al., 1997).

#### Polysomnography

In addition to the wrist actigraphy data, an overnight sleep visit will be required to collect the objective sleep data.

#### Physiological indicators

A blood sample will be collected to measure CGRP and PACAP.

### Data management

The following information will be obtained from each subject: demographics, 4-week headache data (pre and posttreatment), pre and posttreatment outcome variables (PGI-I, PGI-S, MIDAS, HADS, PSQI, and GSDS), selected physiological indicators (PSG, CGRP, and PACAP), and pre and posttreatment 7-day wrist actigraphy data (TST, WASO, circadian activity rhythm). The physiological analysis will be performed in the hospital’s laboratory using established laboratory protocols for specimen processing and analysis. Assay preparation will be performed following the manufacturer’s instructions. The sleep data obtained with the watch will be analyzed by using the Action 4 software, and the PSG will be analyzed by using the Crystal software.

### Data analysis

All data analyzes will be conducted using the intent-to-treat principle. When considering the results of multiple comparisons, the Bonferroni correction will be used to correct the significance level. Pre and posttest, the related scales of the experimental and control groups, such as the headache diary, PGI-I, PGI-S, MIDAS, HADS, PSQI, ESS, GSDS, and PSG, wrist actigraphy, and blood test analysis, will be expressed as mean ± standard deviation.

The above-mentioned scales and blood test values of the pre and posttreatment assessments of each patient will be tested with the Sign test to identify any difference before and after the test. Finally, the generalized estimating equation model will be used to evaluate whether the experimental group significantly had reduced pain frequency and severity and improved sleep than the control group to understand the effect of light therapy on migraine.

### Ethical considerations

The study was approved by the Institutional Review Board of Kuang Tien General Hospital (KTGH21002) and registered in the ClinicalTrials.gov (NCT4890691).

Participation in research is voluntary. Completing the data booklets may result in some discomfort as women will reflect on the answers that they will provide; they will be reminded that they may stop at any time. Women wearing the bright light visor may have a slight chance of developing a headache similar to exposure to natural sunlight; if so, participants will be instructed to use the 8,000 lux instead of 12,000 lux. During the weekly follow-up phone call, participants will be asked if they have any unusual symptoms. If yes, this will be discussed with the Co-PI, who is a neurologist, and, if any adverse symptoms are noted, the participant will be requested to discontinue the light treatment. All participants may choose to terminate their participation at any time without incurring any adverse consequences.

### Discussion

Although there have been considerable advances in the treatment of migraine in the last decade, migraine remains one of the debilitating chronic pain conditions and many patients continue to experience substantial pain and disability, despite taking multiple drugs. Patient and clinician interests in non-pharmacological treatments remain strong (Robbins, 2021).

Migraine and sleep have drawn great attention due to their strong, bidirectional, and complex clinical relationship (Tiseo, Vacca, Felbush, Filimonova, Gai, Glazyrina, Hubalek, Marchenko, Overeem, Piroso, Tkachev, Martelletti, Sacco and European Headache Federation School of Advanced 2020). The hypothalamus involved in homeostatic regulation, including pain processing and sleep–wake cycle regulation, is also a key integrator of circadian entrainment to light and has emerged as a key brain area in migraine (Schulte and May, 2016). Although clinical evidence has shown that light therapy can stabilize sleep architecture, its beneficial effects in migraine patients with sleep disturbance remain to be elucidated.

To date, few studies have tested an intervention designed to prevent migraine by improving sleep. This protocol provides a detailed overview of the design and implementation of a randomized, double-blind, placebo-controlled clinical trial comparing the effects between green light for the treatment group and red light for the control group in migraine patients with sleep disturbance. It is anticipated that this study will provide preliminary support for the use of light therapy in conjunction with sleep hygiene intervention for reducing women’s migraine attacks by improving sleep. This study will provide the basis for further development of nonpharmaceutical interventions for sleep to prevent migraine attacks in various populations and will have direct clinical applicability by adding an important primary intervention for the clinical care of migraine patients.

## Ethics statement

The studies involving human participants were reviewed and approved by Institutional Review Board of Kuang Tien General Hospital (KTGH21002). The patients/participants provided their written informed consent to participate in this study.

## Author contributions

T-HL, C-CY, S-YL, and C-PY were responsible for the study concept and research design, and study design modification. T-HL, C-CY, S-YL, C-MC, and C-PY were also responsible for drafting and revising the manuscript. C-MC, C-YW, and C-PY modified the study design and revised the manuscript. T-HL, C-CY, S-YL, C-MC, C-YW, and C-PY contributed to the collection, analysis, and interpretation of data and revision of the manuscript. All authors have read and approved the final version of the manuscript.

## Funding

This study was supported by Hungkuang University and Kuang Tien General Hospital (HK-KTOH-110-03), Taichung, Taiwan.

## Conflict of interest

The authors declare that the research was conducted in the absence of any commercial or financial relationships that could be construed as a potential conflict of interest.

## Publisher’s note

All claims expressed in this article are solely those of the authors and do not necessarily represent those of their affiliated organizations, or those of the publisher, the editors and the reviewers. Any product that may be evaluated in this article, or claim that may be made by its manufacturer, is not guaranteed or endorsed by the publisher.

## References

[ref1] American Headache Society (2019). The American headache society position statement on integrating new migraine treatments into clinical practice. Headache 59, 1–18. doi: 10.1111/head.13456. 30536394

[ref2] Amin F. M. Hougaard A. Magon S. Asghar M. S. Ahmad N. N. Rostrup E. . (2016). Change in brain network connectivity during PACAP38-induced migraine attacks: a resting-state functional MRI study. Neurology 86, 180–187. doi: 10.1212/WNL.0000000000002261 26674334

[ref3] Ancoli-Israel S. Clopton P. Klauber M. R. Fell R. Mason W. (1997). Use of wrist activity for monitoring sleep/wake in demented nursing-home patients. Sleep 20, 24–27. doi: 10.1093/sleep/20.1.24, PMID: 9130330 PMC2762477

[ref4] Artemenko A. R. Filatova E. Vorobyeva Y. D. Do T. P. Ashina M. Danilov A. B. (2022). Migraine and light: a narrative review. Headache 62, 4–10. doi: 10.1111/head.14250 35041220

[ref5] Ashina M. Amin F. M. Kokturk P. Cohen J. M. Konings M. Tassorelli C. . (2021a). PEARL study protocol: a real-world study of fremanezumab effectiveness in patients with chronic or episodic migraine. Pain Manage. 11, 647–654. doi: 10.2217/pmt-2021-0015 34105377

[ref6] Ashina M. Katsarava Z. Do T. P. Buse D. C. Pozo-Rosich P. Ozge A. . (2021b). Migraine: epidemiology and systems of care. Lancet 17, 1485–1495. doi: 10.1016/S0140-6736(20)32160-7 33773613

[ref7] Baksa D. Gecse K. Kumar S. Toth Z. Gal Z. Gonda X. . (2019). Circadian variation of migraine attack onset: a review of clinical studies. Biomed. Res. Int. 2019:4616417. doi: 10.1155/2019/4616417 31534960 PMC6732618

[ref8] Brandes J. L. (2006). The influence of estrogen on migraine: a systematic review. JAMA 295, 1824–1830. doi: 10.1001/jama.295.15.1824 16622144

[ref9] Burkhalter H. Wirz-Justice A. Denhaerynck K. Fehr T. Steiger J. Venzin R. M. . (2015). The effect of bright light therapy on sleep and circadian rhythms in renal transplant recipients: a pilot randomized, multicentre wait-list controlled trial. Transpl. Int. 28, 59–70. doi: 10.1111/tri.12443, PMID: 25182079

[ref10] Burstein R. Noseda R. Borsook D. (2015). Migraine: multiple processes, complex pathophysiology. J. Neurosci. 29, 6619–6629. doi: 10.1523/JNEUROSCI.0373-15.2015 PMC4412887 25926442

[ref11] Buysse D. J. Reynolds C. F. 3rd Monk T. H. Berman S. R. Kupfer D. J. (1989). The Pittsburgh sleep quality index: a new instrument for psychiatric practice and research. Psychiatry Res. 28, 193–213. doi: 10.1016/0165-1781(89)90047-4, PMID: 2748771

[ref12] Faulkner S. M. Bee P. E. Meyer N. Dijk D. J. Drake R. J. (2019). Light therapies to improve sleep in intrinsic circadian rhythm sleep disorders and neuro-psychiatric illness: a systematic review and meta-analysis. Sleep Med. Rev. 46, 108–123. doi: 10.1016/j.smrv.2019.04.012, PMID: 31108433

[ref13] Guy W. (1993). *ECDEU Assessment Manual for Psychopharmacology*. US Department of Health Education, and Welfare Public Health Service, Alcohol, Drug, Abuse, and Mental Health Administration, National Institute of Mental Health Psychopharmacology Research Branch, Division of Extramural Research Programs.

[ref14] Jean-Louis G. von Gizycki H. Zizi F. Fookson J. Spielman A. Nunes J. . (1996). Determination of sleep and wakefulness with the actigraph data analysis software (ADAS). Sleep 19, 739–743. 9122562

[ref15] Johns M. W. (1991). A new method for measuring daytime sleepiness: the Epworth sleepiness scale. Sleep 14, 540–545. doi: 10.1093/sleep/14.6.540 1798888

[ref16] Lee K. A. (1992). Self-reported sleep disturbances in employed women. Sleep 15, 493–498. doi: 10.1093/sleep/15.6.493 1475563

[ref17] Lee S. Y. (2007). Validating the general sleep disturbance scale among Chinese American parents with hospitalized infants. J. Transcult. Nurs. 18, 111–117. doi: 10.1177/1043659606298502, PMID: 17416712

[ref18] Lee K. A. DeJoseph J. F. (1992). Sleep disturbances, vitality, and fatigue among a select group of employed childbearing women. Birth 19, 208–213. doi: 10.1111/j.1523-536X.1992.tb00404.x, PMID: 1472269

[ref19] Lindskov F. O. Iversen H. K. West A. S. (2022). Clinical outcomes of light therapy in hospitalized patients - a systematic review. Chronobiol. Int. 39, 299–310. doi: 10.1080/07420528.2021.1993240 34727798

[ref20] McCurry S. M. Logsdon R. G. Vitiello M. V. Teri L. (2004). Treatment of sleep and nighttime disturbances in Alzheimer’s disease: a behavior management approach. Sleep Med. 5, 373–377. doi: 10.1016/j.sleep.2003.11.003, PMID: 15222994

[ref21] Noseda R. Burstein R. (2013). Migraine pathophysiology: anatomy of the trigeminovascular pathway and associated neurological symptoms, CSD, sensitization and modulation of pain. Pain 154, S44–S53. doi: 10.1016/j.pain.2013.07.021 23891892 PMC3858400

[ref22] Robbins M. S. (2021). Diagnosis and Management of Headache: a review. JAMA 11, 1874–1885. doi: 10.1001/jama.2021.1640 33974014

[ref23] Schulte L. H. May A. (2016). The migraine generator revisited: continuous scanning of the migraine cycle over 30 days and three spontaneous attacks. Brain 139, 1987–1993. doi: 10.1093/brain/aww097, PMID: 27190019

[ref24] Silberstein S. D. (2008). Recent developments in migraine. Lancet 372, 1369–1371. doi: 10.1016/S0140-6736(08)61569-X 18940454

[ref25] Steiner T. J. Stovner L. J. Vos T. Jensen R. Katsarava Z. (2018). Migraine is first cause of disability in under 50s: will health politicians now take notice? J. Headache Pain 19:17. doi: 10.1186/s10194-018-0846-2 29468450 PMC5821623

[ref26] Stewart W. F. Lipton R. B. Kolodner K. Liberman J. Sawyer J. (1999). Reliability of the migraine disability assessment score in a population-based sample of headache sufferers. Cephalalgia 19, 107–114. doi: 10.1046/j.1468-2982.1999.019002107.x, PMID: 10214536

[ref27] Stovner L. Hagen K. Jensen R. Katsarava Z. Lipton R. Scher A. . (2007). The global burden of headache: a documentation of headache prevalence and disability worldwide. Cephalalgia 27, 193–210. doi: 10.1111/j.1468-2982.2007.01288.x, PMID: 17381554

[ref28] Tiseo C. Vacca A. Felbush A. Filimonova T. Gai A. Glazyrina T. . (2020). Migraine and sleep disorders: a systematic review. J. Headache Pain 21:126. doi: 10.1186/s10194-020-01192-5 33109076 PMC7590682

[ref29] Vgontzas A. Pavlovic J. M. (2018). Sleep disorders and migraine: review of literature and potential pathophysiology mechanisms. Headache 58, 1030–1039. doi: 10.1111/head.13358, PMID: 30091160 PMC6527324

[ref30] Wang S. J. Fuh J. L. Young Y. H. Lu S. R. Shia B. C. (2000). Prevalence of migraine in Taipei, Taiwan: a population-based survey. Cephalalgia 20, 566–572. doi: 10.1046/j.1468-2982.2000.00085.x, PMID: 11075840

[ref31] Zigmond A. S. Snaith R. P. (1983). The hospital anxiety and depression scale. Acta Psychiatr. Scand. 67, 361–370. doi: 10.1111/j.1600-0447.1983.tb09716.x 6880820

